# Risk of breast cancer-related death in women with a prior cancer

**DOI:** 10.18632/aging.102984

**Published:** 2020-04-06

**Authors:** Fei Ji, Ci-Qiu Yang, Xiao-Ling Li, Liu-Lu Zhang, Mei Yang, Jie-Qing Li, Hong-Fei Gao, Teng Zhu, Min-Yi Cheng, Wei-Ping Li, Si-Yan Wu, Ai-Ling Zhong, Kun Wang

**Affiliations:** 1 Department of Breast Cancer, Cancer Center, Guangdong Provincial People's Hospital, Guangdong Academy of Medical Sciences, Guangzhou 510080, P.R. China; 2 Department of Operation Room, Guangdong Provincial People's Hospital, Guangdong Academy of Medical Sciences, Guangzhou 510080, P.R. China; 3 School of Medicine, South China University of Technology, Guangzhou 510006, P.R. China

**Keywords:** second primary malignancy, breast cancer, risk factor, survival

## Abstract

The overall risk of developing a second primary cancer is increasing. The purpose of this study was to analyze the survival of patients with breast cancer diagnosed after a prior cancer and identify risk factors of breast cancer death in this population. Using the SEER database, we identified 1,310 woman diagnosed with breast cancer between 2010 and 2015 after a prior cancer as the primary cohort. Clinicopathological characteristics were compared using the Student *t*-test and chi-square test. Fine and Gray’s regression was used to evaluate the effect of treatments on breast cancer death. After propensity score matching (PSM), 9,845 pairs of patients with breast cancer as the prior or second cancer diagnosed between 2010 and 2011 were included as a second cohort. PSM-adjusted Kaplan-Meier and Cox hazards models were used to evaluate the impact of prior cancer on survival. The results showed that survivors of gynecologic cancers (e.g., ovarian cancer) had a higher risk of developing breast cancer than survivors of gastrointestinal and urinary tract cancers. More patients died of breast cancer than of prior urinary cancer (53.3% vs. 40%, P < 0.05) and melanoma (66.7% vs. 33.3%, P < 0.05). The ratio of breast cancer deaths to prior cancer deaths was significantly higher in patients with diagnoses interval ≥ 3 years than in those with the interval < 3 years (2.67 vs. 0.69, P < 0.001). Breast cancer-specific survival and overall survival rates were significantly lower in women with breast cancer as the second primary cancer than in those with breast cancer as the prior cancer, especially among hormone receptor-positive women. However, breast cancer treatment decreased the risk of breast cancer -specific death (hazard ratio = 0.695, 95% confidence interval: 0.586–0.725, P < 0.001). Breast cancer patients with prior cancers must be carefully considered for clinical trials.

## INTRODUCTION

Due to the advances in the detection of early-stage cancers and cancer treatment, the population of cancer survivors has increased by approximately 4 folds in the United States in the past 30 years [1–3]. Almost two-thirds of cancer survivors live more than 5 years after the initial diagnosis, which increases the risk of developing a second primary malignancy (SPM) [4–8]. A SPM is defined as a cancer which arises independently in a new organ or tissue at least 2 months after the diagnosis of the prior primary cancer. Over 10% of younger adult cancer patients and around 25% of older adult cancer patients have been diagnosed with a SPM [9, 10]. It has been reported to be associated with genetic susceptibility [11, 12], the carcinogenic adverse effects of cancer treatment [13], and behavioral risk factors such as smoking and alcohol intake [14–18].

Many studies have examined the risk of a SPM in patients with different types of prior cancer, such as lung, testis, head and neck, and thyroid cancers [19–21]. The prognosis of patients with stage IV lung cancer as a SPM was not affected by a prior cancer [22]. The prevalence of colorectal adenomas in breast cancer survivors is similar to that in patients with single colorectal adenomas [23]. Garg et al. [24] reported that early diagnosis and the absence of recurrence in patients with prior breast cancer who had abdominal carcinomatosis were significantly associated with the development of ovarian/peritoneal cancer as a SPM. Prior breast cancer and tamoxifen exposure did not affect the prognosis of women with uterine papillary serous carcinoma as a SPM [25]. However, the risk of breast cancer as a SPM in patients with a prior cancer and the cancer-specific survival for these patients are not known.

Thus, in the present study, we used data from the Surveillance, Epidemiology, and End Results (SEER) database to analyze the outcomes of patients who developed breast cancer as a SPM. This information may help guide long-term surveillance strategies for patients.

## RESULTS

### Baseline characteristics of the primary cohort

The median (interquartile range, IQR) age at diagnosis of the prior cancer was 66 (20–96) years, and that of subsequent breast cancer was 68 (20–99) years. Most patients were Caucasian (1,097, 83.8%), and only a few (111, 8.5%) were black. Overall, 920 (70.2%) patients had their prior cancers diagnosed at TNM stage I-II, and 1094 (83.5%) had their breast cancers diagnosed at TNM stage I-II. The median (IQR) interval between 2 diagnoses was 17 (2–71) months. By the end of 2017, the patients were followed up for a median of 17 months (IQR, 0-69 months), and 219 (16.7%) patients died during follow-up: 153 (69.9%) died of cancer, and 66 (30.1%) died of other causes (Table 1).

**Table 1:** Summary description of demographic and clinical factors.

**At prior cancer diagnosis**			**At breast cancer diagnosis**	
**Variable**	**Value**		**Variable**	**Value**
Age, years			Age, years	
Mean(range)	65.8(20-96)		Mean(range)	67.8(20-99)
Median(IQR)	66		Median(IQR)	68
Race,n(%)			Race,n(%)	
White	1098(83.8%)		White	1098(83.8%)
Black	111(8.5%)		Black	111(8.5%)
Other	101(7.7%)		Other	101(7.7%)
Marital status			Marital status	
Married	997(76.1%)		Married	916(69.9%)
Unmarried	313(23.9%)		Unmarried	394(30.1%)
TNM stage,n(%)			TNM stage,n(%)	
I-II	919(70.2%)		I-II	1094(83.5%)
III-IV	391(29.8%)		III-IV	216(16.5%)
Interval between diagnoses, months			Follow up from BC diagnosis to death or end of study(months)	
Mean(range)	21.64		Mean(range)	20.96
Median(IQR)	17(2–71)		Median(IQR)	17(0-69)

### Breast cancer-related deaths in the primary cohort

**Figure 1:**
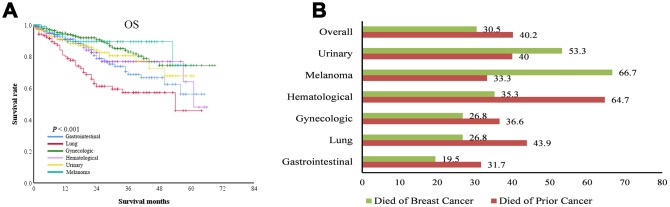
**Overall survival (OS) of patients with breast cancer as a second primary cancer.** (**A**) OS curves of patients with different types of prior cancer. (**B**) the percentage of deaths related to breast cancer or prior cancer among patients with different types of prior cancer. For some types of prior cancer, breast cancer resulted in more deaths than the prior cancer.

OS varied significantly among patients with different types of prior cancer (P < 0.001), and patients with prior lung cancer had the shortest OS (Figure 1A). We stratified patients by death of prior cancer and death of breast cancer as an SPM. Overall, 30.5% of patients died of breast cancer, and 40.2% of patients died of the prior cancer. The breast cancer-related death rate was the lowest (19.5%) in patients with prior gastrointestinal cancer and the highest (66.7%) in patients with prior melanoma. Breast cancer caused more deaths than prior urinary cancer (53.3% vs. 40%) and melanoma (66.7% vs. 33.3%), but caused less deaths than prior lung cancer (26.8% vs. 43.9%), hematological cancer (35.3% vs. 64.7%), and gynecologic cancer (26.8% vs. 36.6%) (all P < 0.05) (Figure 1B). The ratio of breast cancer deaths to prior cancer deaths was markedly higher in patients with diagnoses interval ≥ 3 years than in those with the interval < 3 years (2.67 vs. 0.69, P < 0.001).

### Associations of demographic and clinicopathologic factors with breast cancer deaths in the primary cohort

**Figure 2:**
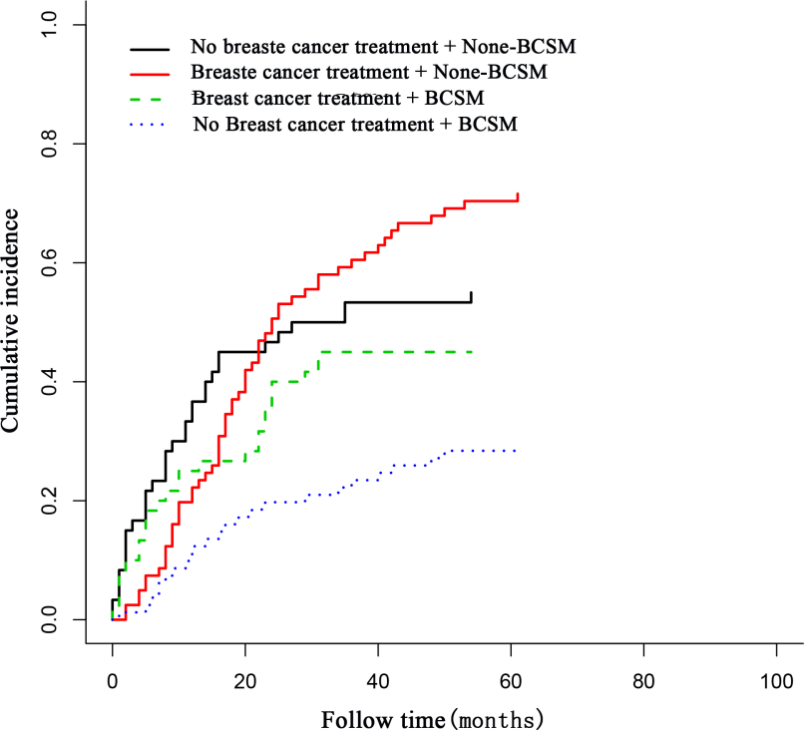
**The association of breast cancer treatment with breast cancer deaths in patients with breast cancer as a second primary cancer.** Breast cancer treatment was associated with decreased breast cancer deaths in these patients.

The rates of high-grade disease (32.3% vs. 23.1%, P < 0.001), T3–4/N0/M0 disease (11.3% vs. 4.4%, P = 0.014), and distant disease (33.9% vs. 27.5%, P = 0.023) of breast cancer were significantly higher in patients who died of breast cancer than in those who died of a prior cancer, and the rates of metastasis of prior cancer (11.3% vs. 25.3%, P = 0.011) and administration of breast cancer treatment (37.1% vs. 63.4%, P = 0.018) were significantly lower in patients who died of breast cancer. Patients who died of breast cancer had a longer diagnosis interval (17.0 vs. 11.4 months, P = 0.040) and were older (73.5 vs. 69.1 years, P = 0.035) than those who died of a prior cancer (Table 2). Breast cancer treatment was associated with a decreased risk of breast cancer-specific mortality (BCSM) (hazard ratio = 0.695, 95% confidence interval [CI] = 0.586–0.725, P < 0.001), but not associated with a decreased risk of non-BCSM (Figure 2).

**Table 2:** Clinical and demographic factors associated with breast cancer death vs prior malignancy death.

**Character**	**Died from Breast cancer**	**Died from prior cancer**	**P value**
Number of patients(%)	62	91	
High grade	32.3%	23.1%	<0.001
Breast cancer stage T3-4, N0/M0	11.3%	4.4%	0.014
Tx/N1-3/Mx or Tx/Nx/M1 Breast cancer	33.9%	27.5%	0.023
Tx/N1/Mx or Tx/Nx/M1 Prior cancer	11.3%	25.3%	0.011
Breast cancer treated	37.1%	63.4%	0.018
Interval between diagnoses, months	17.0	11.4	0.04
Age at Breast cancer diagnosis, years	73.5	69.1	0.03

### Risk of breast cancer-related deaths among patients with different types of prior cancer in the primary cohort

**Figure 3:**
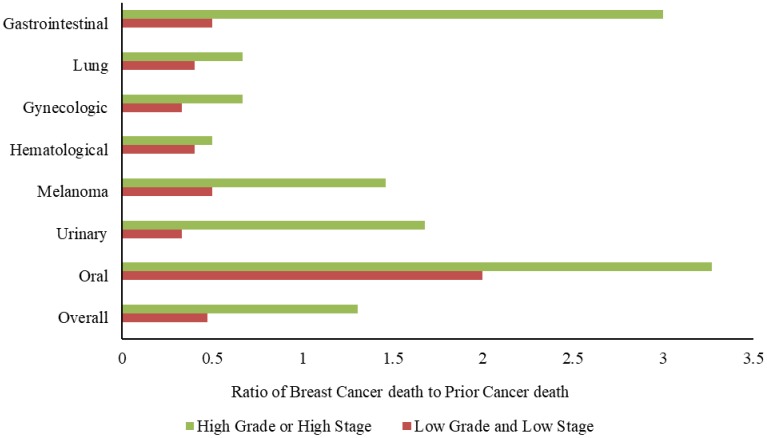
**Ratio of breast cancer deaths to prior cancer deaths among patients with different types of prior cancer.** Patients with prior gastrointestinal cancer, melanoma, and urinary tract cancer were more likely to die of breast cancer when they had high-grade or stage cT3–T4/N0/M0 breast cancer, but were more likely to die of prior cancer when they had low-grade and stage cT1–2/N0/M0 breast cancer.

To further determine the risk of breast cancer-related deaths among patients with different types of prior cancer, we calculated the ratio of breast cancer deaths to prior cancer deaths in patients with low-grade and low stage(stage cT1–2/N0/M0) and in those with high-grade or high stage(stage cT3–T4/N0/M0) cancer (Figure 3). Overall, the ratio was 0.47 in patients with low-grade and stage cT1–2/N0/M0 disease and 1.31 in patients with high-grade or stage cT3–T4/N0/M0 breast cancer, indicating that the risk of breast cancer deaths was much higher than that of prior cancer deaths in the latter group of patients. In total, 51.8% of patients with high-grade or stage cT3–4/N0/M0 breast cancer were likely to die of breast cancer, whereas only 34.2% of patients with low-grade and low stage disease died of breast cancer (P < 0.001). Interestingly, patients with prior oral cancer were more likely to die of breast cancer, whereas those with prior lung, hematological, and gynecological cancers were more likely to die of the prior cancer. However, taking tumor grade and stage into consideration, patients with prior gastrointestinal cancer, melanoma, and urinary tract cancer were more likely to die of breast cancer when they had high-grade or stage cT3–T4/N0/M0 breast cancer, but were more likely to die of prior cancer when they had low-grade and cT1–2/N0/M0 breast cancer.

### Survival of patients with breast cancer as the prior cancer or subsequent primary cancer in the second cohort

**Figure 4:**
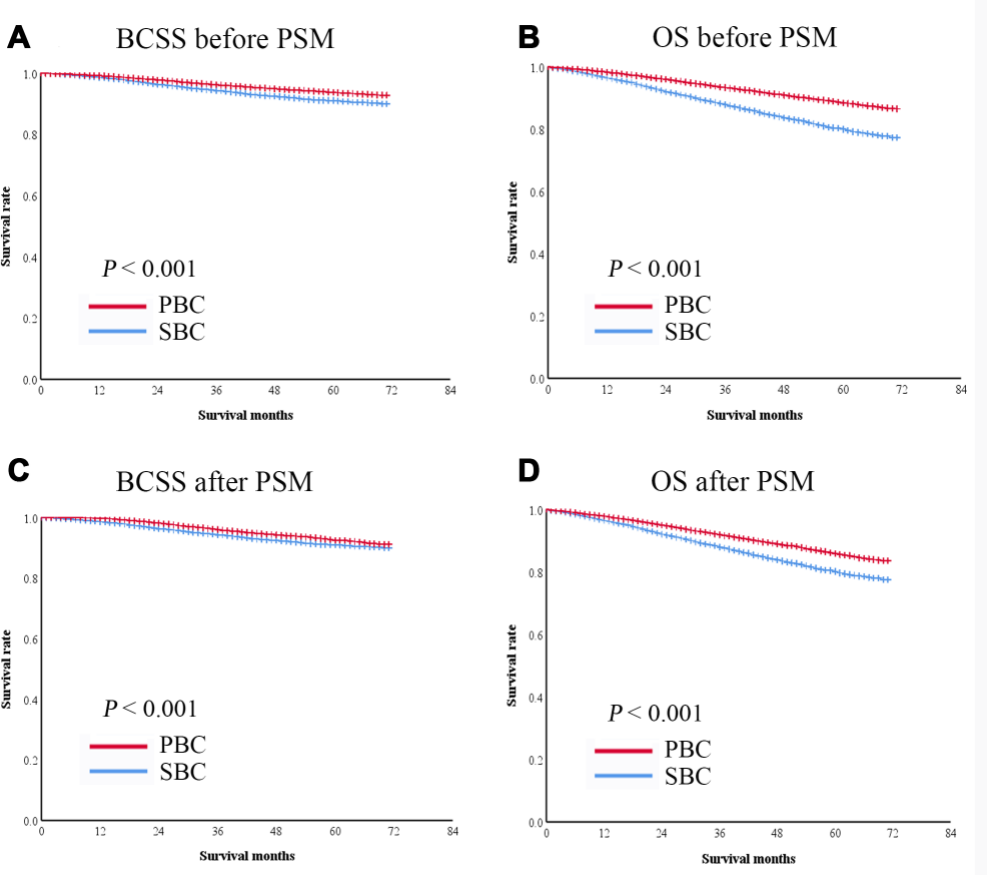
**Kaplan-Meier survival curves of patients with breast cancer as the second primary cancer or the prior cancer.** (**A**) BCSS was significantly shorter in patients with breast cancer as the second primary cancer than in those with breast cancer as the prior cancer in the entire cohort. (**B**) OS was significantly shorter in patients with breast cancer as the second primary cancer than in those with breast cancer as the prior cancer in the entire cohort. After PSM, both BCSS (**C**) and OS (**D**) were significantly lower in patients with breast cancer as the second primary cancer than in those with breast cancer as the prior cancer.

In the second cohort, 63,761 patients had breast cancer as their only cancer (primary breast cancer, PBC), and 9,955 had breast cancer as the second primary cancer (subsequent breast cancer, SBC). The 5-year BCSS and OS rates were significantly lower in patients with SBC than in those with PBC (BCSS: 91.0% vs. 94.0%, P < 0.001, Figure 4A; OS: 91.0% vs. 93.8%, P < 0.001, Figure 4B).

A 1:1 PSM was performed to balance the baseline characteristics of patients with SBC and PBC. After matching, 9,845 pairs of patients were included in the analysis (Table 3). The survival of the matched groups was in consistent with the survival of the entire cohort, the 5-year BCSS and OS rates were significantly lower in patients with SBC than in those with PBC (BCSS: 91.2% vs. 93.6%, P < 0.001, Figure 4C; OS: 80.5% vs. 86.1%, P < 0.001, Figure 4D).

**Table 3:** Baseline characteristics of patients with PBC or SBC from the SEER database 2010–2011.

**Variables**	**Data before PSM**		**P value**	**Data after PSM**		**P value**
	**PBC (63761)**	**SBC (9955)**		**PBC (9845)**	**SBC (9845)**	
**Age(y), %**			<0.001			>0.99
18-45	9485	615		600	600	
46-65	32848	4014		3995	3995	
≥65	21428	5326		5250	5250	
**Race, %**			<0.001			>0.99
White	50893	8388		8347	8347	
Black	6641	895		854	854	
Others	6227	672		644	644	
**Marital status, %**			<0.001			>0.99
Married	38474	5392		5340	5340	
Not married	25287	4563		4505	4505	
**Tumor differentiation, %**			<0.001			>0.99
I–II	42439	7066		6984	6984	
III–IV	21322	2889		2861	2861	
**TNM Stage, %**			<0.001			>0.99
I	31769	5963		5897	5897	
II–III	31992	3992		3948	3948	
**Breast subtype, %**			<0.001			>0.99
HER2^-^/HR^+^	46913	7508		7469	7469	
HER2^+^/HR^+^	6377	799		779	779	
HER2^+^/HR^-^	2761	373		354	354	
Triple-negative	7710	1275		1243	1243	
**Surgery, %**			<0.001			>0.99
Lumpectomy	37756	4679		4644	4644	
Mastectomy	26005	5276	<0.001	5201	5201	
**Radiation, %**					>0.99
Yes	27815	6298		6221	6221	
No/ Unknown	35946	3657	<0.001	3624	3624	
**Chemotherapy, %**						>0.99
Yes	36349	6847		6797	6797	
No/unknown	27412	3108		3048	3048	

### Survival of patients with hormone receptor (HR)-positive breast cancer as the prior cancer or second primary cancer

**Figure 5:**
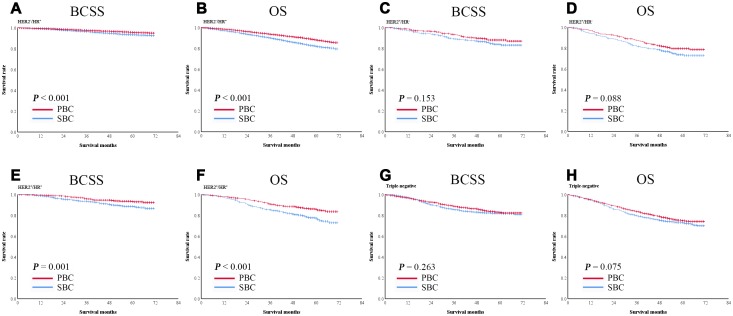
**Kaplan-Meier survival curves of patients with breast cancer as the second primary cancer or the prior cancer and with different hormone receptor statuses.** Breast cancer as the second primary cancer was significantly associated with shorter BCSS and OS in hormone receptor-positive subgroups (**A**–**H**).

Taking hormone receptor statuses into consideration, breast cancer as the subsequent primary cancer (SBC) was significantly associated with short BCSS and OS in HER2-/HR+ patients (both P < 0.001, Figure 5A, 5B) and HER2+/HR+ patients (P = 0.001and P < 0.001) (Figure 5E, 5F). However, no such associations were observed in HER2+/HR- and triple-negative patients (Figure 5C, 5D, 5G, 5H).

### Factors associated with survival of patients with breast cancer as the primary cancer or subsequent breast cancer

We used univariate and multivariate analyses to determine clinicopathological factors which associated with survival. Univariate analysis showed that age, race, marital status, tumor differentiation, TNM stage, breast cancer subtype, breast cancer as the prior or second primary cancer (PBC vs SBC), surgery, radiotherapy, and chemotherapy were significant risk factors for both BCSS and OS (all P < 0.001) (Table 4). Cox regression multivariate analysis indicated that marital status, tumor differentiation, TNM, breast cancer subtype, breast cancer as the primary or subsequent breast cancer (PBC vs SBC), and radiotherapy were independent prognostic factors for both BCSS and OS, race was an independent prognostic factor for BCSS, and age and chemotherapy were independent prognostic factors for OS (all P < 0.05) (Table 4).

**Table 4:** Univariate and Multivariate Cox regression model analysis of BCSS and OS.

**Category**	**BCSS**					**OS**			
	**5 years**	**Univariate**	**HR (95% CI)**	**Multivariate**		**5 years**	**Univariate**	**HR (95% CI)**	**Multivariate**
**Age**		P<0.001					P<0.001		
18-45	90%		Reference			87.6%		Reference	
45-65	93.6%		0.899 (0.732-1.104)	0.309		90.7%		0.883 (0.737-1.059)	0.179
>65	91.9%		1.368 (1.118-1.674)	0.002		77.3%		2.167 (1.817-2.586)	P<0.001
**Race**		P<0.001					P<0.001		
White	92.3%		Reference			83.5%		Reference	
Black	86.2%		1.261 (1.083-1.469)	0.003		77.1%		1.155 (1.034-1.291)	0.011
Other	94.5%		0.690 (0.535-0.890)	0.004		88.4%		0.749 (0.633-0.886)	0.001
**Marital status**		P<0.001					P<0.001		
Married	93.9%		Reference			88.3%		Reference	
Not married	90.7%		1.307 (1.171-1.459)	P<0.001		77.4%		1.606 (1.492-1.728)	P<0.001
**Tumor differentiation**		P<0.001					P<0.001		
I–II	95.6%		Reference			86.1%		Reference	
III–IV	84.8%		2.143 (1.891-2.429)	P<0.001		76.4%		1.479 (1.361-1.6078)	P<0.001
**TNM Stage**		P<0.001					P<0.001		
I	97.0%		Reference			88.9%		Reference	
II–III	85.4%		4.438 (3.903-5.045)	P<0.001		74.8%		2.553 (2.369-2.752)	P<0.001
**Breast subtype**		P<0.001					P<0.001		
HER2^-^/HR^+^	94.6%		Reference			85.3%		Reference	
HER2^+^/HR^+^	90.9%		1.246 (1.031-1.505)	0.023		76.5%		1.283 (1.130-1.458)	P<0.001
HER2^+^/HR^-^	85.9%		1.517 (1.207-1.096)	P<0.001		81.8%		1.557 (1.314-1.845)	P<0.001
Triple-negative	82.6%		2.209 (1.769-2.327)	P<0.001		74.2%		1.711 (1.545-1.894)	P<0.001
**Diagnosis**		P<0.001					P<0.001		
PBC	93.7%		Reference			86.1%		Reference	
SBC	91.2%		1.444 (1.297-1.607)	P<0.001		80.5%		1.493 (1.392-1.602)	P<0.001
**Surgery**		P<0.001					P=0.272		
Lumpectomy	94.0%					83.6%			
Mastectomy	91.1%					83.0%			
**Radiation**		P<0.001					P<0.001		
Yes	94.4%		Reference			88.9%		Reference	
No/ Unknown	91.3%		1.517 (1.349-1.707)	P<0.001		80.0%		1.768 (1.630-1.918)	P<0.001
**Chemotherapy**		P<0.001					P<0.001		
Yes	89.0%					85.3%		Reference	
No/unknown	94.1%					82.4%		1.424 (1.300-1.559)	P<0.001

**Figure 6:**
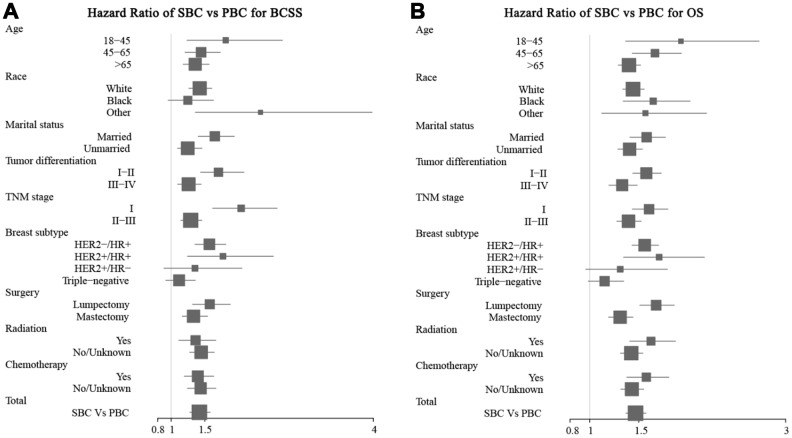
**Forest plot of the hazard ratios for survival of patients with breast cancer as the second primary cancer or the prior cancer.** Breast cancer as the second primary cancer was associated with short BCSS (**A**) and OS (**B**) in most of the subgroups.

As shown on Figure 6, compared with breast cancer as the primary cancer (PBC), breast cancer as the SBC was associated with shorter BCSS when the age at diagnosis was > 65 years (hazard ratio = 1.36, 95% CI: 1.18–1.81), with grade III and IV tumors (hazard ratio = 1.26, 95% CI: 1.10–1.45), with triple-negative disease (hazard ratio = 1.12, 95% CI: 0.919–1.36), mastectomy (hazard ratio = 1.33, 95% CI: 1.16–1.54), radiotherapy (hazard ratio = 1.36, 95% CI: 1.11–1.50), and chemotherapy (hazard ratio = 1.40, 95% CI: 1.19–1.63) and was associated with shorter OS when the age at diagnosis was > 65 years (hazard ratio = 1.40, 95% CI: 1.29–1.52), with grades III and IV tumors (hazard ratio = 1.33, 95% CI: 1.20–1.49), HER2+/HR- disease (hazard ratio = 1.31, 95% CI: 0.96–1.79), triple-negative breast cancer (hazard ratio = 1.15, 95% CI: 0.99–1.35), and mastectomy (hazard ratio = 1.31, 95% CI: 1.19–1.44). These data suggest that breast cancer as the second primary cancer affects the prognosis of patients who have had a prior cancer.

## DISCUSSION

The cancer survivor population is rapidly growing, and subsequently the number of patients with multiple primary or multi-organ cancers is also increasing [26]. In the US, the cancer survivor population presented a 2% annual increase, and approximately 18% of cancer cases were developed after the prior cancer according to SEER data [27]. The risk of developing an SPM among cancer survivors was reported to be as high as that in the general population [28]. The risk of breast cancer as a second primary cancer is especially high [29]. However, the clinicopathological characteristics and survival of patients with this disease remain largely unknown.

In this population-based study, we found that gynecologic cancers, gastrointestinal cancer, urinary tract cancers, hematological cancers, lung cancer, and melanoma were the most common types of prior cancer. Survivors of gynecologic cancers such as ovarian cancer tended to have a high risk of developing breast cancer as their second cancer. This may be explained by that hereditary breast cancer and ovarian cancer are the most common cancers with genetic predisposition. *BRCA1* and/or *BRCA2* mutations are frequently detected in both cancer types. The risk of developing breast cancer is increased among patients with prior ovarian cancer harboring *BRCA* mutations. The 10-year occurrence rates of breast cancer among patients with prior ovarian cancer were 12% in *BRCA1* mutation carriers and 2% in *BRCA2* mutation carriers [30, 31]. Therefore, annual screening with breast magnetic resonance imaging is recommended for patients with *BRCA1* and *BRCA2* mutations. In the present study, 30.5% of women with breast cancer as a SPM died of breast cancer, and 40.2% of them died of prior gynecologic cancer. However, among patients with different types of prior cancer, those with prior lung cancer had the shortest OS, those with prior oral cancer were more likely to die of breast cancer regardless of tumor grade and stage, and patients with gastrointestinal cancer, melanoma, and urinary tract cancer were more likely to die of breast cancer when they had high-grade or advanced stage breast cancer. In the whole cohort, 51.8% of patients with high-grade or stage cT3–4/N0/M0 breast cancer died of breast cancer. These data indicate that breast cancer as the second primary cancer remains a main cause of death in women with a prior cancer.

Our results showed that breast cancer as the second primary cancer was associated with short survival. The 5-year BCSS and OS rates were significantly lower in patients with breast cancer as the second primary cancer than in those with breast cancer as the prior cancer in both the standard and PSM analyses, these results are similar to those observed by Zhou et al. [32]. A subgroup survival analysis also showed that HER2−/HR+ and HER2+/HR+ patients with breast cancer as the second primary cancer had shorter BCSS and OS than other subgroup. As such, breast cancer patients with prior cancers must be carefully considered for clinical trials. Univariate and multivariate analyses indicated that breast cancer as the second primary cancer was an independent risk factor for BCSS and OS. The high occurrence and death rates of breast cancer in cancer survivors may likely be attributed to *BRCA1* and *BRCA2* mutations as well as chemotherapy and radiotherapy for prior cancer. Patients who received underwent chemotherapy for prior cancer may not respond well to systemic treatment for their second primary cancer due to decreased efficacy and tolerability after prior systemic treatment [33, 34]. Interestingly, Fine and Gray’s regression analysis showed that breast cancer treatment was associated with a decreased risk of BCSM. The ratio of breast cancer deaths to prior cancer deaths was significantly higher in patients with diagnoses interval ≥ 3 years than in those with the interval < 3 years. Although patients’ breast cancer as the second primary cancer had short survival, breast cancer treatment can still prolong survival.

Some limitations in the present study need to be mentioned. First, as a retrospective study, selection bias was inevitable. Although we performed PSM to minimize such bias, a PSM analysis is also vulnerable to hidden biases, and residual confounding factors could not be entirely ruled out. Second, as the data were retrieved from the SEER database, we could not control treatment-related factors of the prior cancer, which may alter findings related to second cancer incidence and survival. Therefore, further studies with more data are required to validate our findings.

## CONCLUSIONS

Survivors of gynecologic cancers such as ovarian cancer tend to have a high risk of breast cancer. Breast cancer as a second primary cancer, especially locally advanced or high-grade breast cancer, is a significant cause of death in patients with a prior cancer. However, breast cancer treatment decreased the risk of BCSM in these patients. Considering the increases in cancer survivors and deaths related to breast cancer as the second primary cancer, effective detection and treatment strategies is warranted to be investigated in this population.

## MATERIALS AND METHODS

The SEER database is a population-based cancer registry sponsored by the US National Cancer Institute (NCI). The SEER program collects data regarding patient demographic characteristics, cancer incidence, treatment, and survival. The current study included 2 independent patient cohorts derived from the SEER database for separate analyses.

### Primary cohort

The principal analysis of this study used the multiple primary-standard incidence ratio function of SEER*Stat version 8.1.5 software. Using the software to select patients from the registry that covered the 9 regions of the US recognized by the SEER program (covering 9.4% of the US population), we identified 13,011 patients with breast cancer as SPM diagnosed between 2010 and 2015. After excluding patients with bilateral breast cancer, missing information, non-malignant prior neoplasm, multiple malignancies, and diagnosis at autopsy or by death certificate only, 1,310 patients were included in the analysis. The 6 most common types of prior cancer in these 1,310 patients were gynecologic cancer (26.7%) (including ovarian and uterus cancers), gastrointestinal cancer (16.0%) (including gastric, colon, and rectal cancers), urinary tract cancer (11.5%) (including kidney, ureteral, bladder cancers), hematological cancer (10.4%) (including non-Hodgkin lymphoma and leukemia), lung cancer (9.5%), and melanoma (7.9%).

Demographic and clinicopathological data extracted from the SEER database included age, race, marital status, tumor grade, TNM stage, surgical type, and administration of radiotherapy and chemotherapy. Race was divided into white, black, and others. Patients were classified as married or unmarried. The SEER program uses the 7^th^ edition of the American Joint Committee on Cancer (AJCC) TNM staging system. Survival data in the database are presented in the unit of months. We calculated the interval between diagnoses of the prior cancer and breast cancer and the interval from breast cancer diagnosis to death or last follow-up. Each patient was categorized as alive, dead of breast cancer, dead of the prior cancer, or dead of other causes.

Demographic, clinical, and pathological characteristics were compared between patients who died of breast cancer and those who died of the prior cancer using the Student *t* test, and rank-sum and chi-square analyses. The ratio of the percentage of breast cancer deaths to the percentage of prior cancer deaths was calculated for each prior cancer type, stratified by breast cancer grade and TNM stage. Fine and Gray’s competing risk regression model was used to assess the association of breast cancer treatment with breast cancer-specific mortality (BCSM), with death from prior cancer and other causes setting as competing risks. All 2-sided P values < 0.05 were considered significant. Statistical analyses were conducted using R version 3.2.2 software (R Foundation for Statistical Computing, Vienna, Austria).

### Secondary cohort

The secondary cohort included 73,716 patients with stage I-III, histologically confirmed breast cancer identified in the 2010–2011 SEER database, which covered 18 regions of the US (covering 27.8% of the US population). Each patient was categorized as having breast cancer as her only malignancy (primary breast cancer, 63,761 women, 86.5%) or having breast cancer as an SPM (9845 women, 13.5%). Data extracted included age, race, marital status, tumor differentiation, TNM stage, and breast cancer subtype. Patients were assigned to low-, intermediate-, and high-risk groups according to the risk categories of St. Gallen 2007 [35, 36].

The propensity score matching (PSM) method with a ratio of 1:1 was performed to balance the baseline characteristics of patients with breast cancer as the second primary cancer and as the prior cancer [37]. Breast cancer-specific survival (BCSS) was defined as the duration from the diagnosis of breast cancer to death due to breast cancer. Overall survival (OS) was defined as the duration from the diagnosis of breast cancer to death from any cause. Survival between groups was compared using the Kaplan-Meier method. Logistic regression analyses were used to examine the relations between clinicopathological characteristics. A 2-sided P value of < 0.05 was considered significant.
